# Exploratory study of patients’ and carers’ preferences for postdischarge surgical wound monitoring using survey and interviews

**DOI:** 10.1136/bmjopen-2024-087320

**Published:** 2025-01-25

**Authors:** Judith Tanner, Lyn Brierley Jones, Nigel Westwood, Melissa Rochon, Catherine Wloch, Luke J Rogers, Ricky Vaja, Jeremy Dearling, Keith Wilson, Pauline Harrington, Colin S Brown, Gavin Murphy

**Affiliations:** 1 University of Nottingham, Nottingham, UK; 2 School of Health and Life Sciences, Teesside University, Middlesbrough, UK; 3 Patient Researcher, Nottingham, UK; 4 Guy’s and St Thomas’ NHS Foundation Trust, London, UK; 5 UK Health Security Agency, London, UK; 6 Peter Munk Cardiac Surgery Centre, Toronto General Hospital, Toronto, Ontario, Canada; 7 Patient and Public Advisor, King's Lynn, UK; 8 Liverpool Heart and Chest Hospital NHS Foundation Trust, Liverpool, UK; 9 Cardiovascular Sciences, University of Leicester College of Medicine Biological Sciences and Psychology, Leicester, UK

**Keywords:** Infection control, Patient-Centered Care, WOUND MANAGEMENT

## Abstract

**Abstract:**

**Objectives:**

To explore patients’ and carers’ preferences for postdischarge surgical wound monitoring.

**Design:**

Explanatory mixed methods study with an online survey followed by online interviews.

**Setting:**

The online survey was distributed via the Cardiothoracic Interdisciplinary Research Network and cardiac surgery patient and public involvement groups in London and Leicester, UK. Participants were invited to share the survey link with other patients and carers. Interviewees were recruited through the survey.

**Participants:**

Seventy participants completed the survey: 74% patients and 26% carers. A range of ages, sex, ethnicities and geographical locations were represented. Six survey patient participants volunteered to be interviewed.

**Findings:**

Themes identified were the impact on patients of having a surgical site infection, patients’ preferences for postdischarge surgical wound follow-up, access to specialist support, wound monitoring using digital technology and receiving information from the hospital about wounds and wound care. Interviewees described feeling isolated after discharge from hospital and 10% of survey patient respondents, including four of the six interviewees, reported hospital readmissions. Survey respondents’ preferred routes for providing hospitals with wound information were over the telephone (30%), emails (24%), text messages (16%) and photos sent securely (14%). All six interviewees’ preference was for digital approaches using images. Survey respondents were least likely (50%) to reply to questionnaires that required software to be downloaded and installed. Interviewees considered digital wound monitoring to be convenient and the best use of patient and staff resources. A new theme was identified where patients wanted to become more involved in treating their surgical wounds at home.

**Conclusion:**

Experiences described by participants suggests there is a need to improve post-discharge wound monitoring. A new approach should be proactive, ongoing and provide easy access to healthcare services. Digital surgical wound monitoring offers these benefits and is acceptable to patients.

**Trial registration number:**

ISRCTN13950775; Post-results.

**STRENGTHS AND LIMITATIONS OF THIS STUDY**

- To the best of our knowledge, this is the first study to explore patients’ and carers’ preferences for surgical wound monitoring.
- The aim of the survey was to identify issues for further exploration, rather than assign statistical significance and generalisable findings.
- The number of interviews was comparatively small, but the data was substantial and rich in explanatory power.

## Introduction

 Surgical site infections (SSIs) are infections that develop in a patient’s wound site after surgery. They are among the most common healthcare associated infection globally with around 5% of surgical patients estimated to develop an SSI.^1 2^ Infections may be superficial and can be treated relatively quickly with antibiotics, or they can be more severe resulting in hospital readmission, further surgery, reduced quality of life and increased risk of death.^3^ The average estimated cost to the healthcare provider for treating an SSIs in 2021 is £3539, and they are also costly to patients.^4 5^

Most SSIs develop in the early weeks after surgery, usually when patients have been discharged from hospital and are recovering at home.^6^ Early detection of wound complications can prevent problems from worsening;^7^ therefore, wound monitoring during this time is critical.

Usual care following most surgical procedures is to advise patients to contact their general physician (GP) if they have a wound problem; additionally, some patients who have had major procedures may be given an outpatient appointment around 6 weeks after surgery. Additionally, many hospitals participate in SSI surveillance programmes that provide infection rate data which can be used for benchmarking against other hospitals and to make changes in clinical practice to reduce the risk of patients developing SSIs.^8^ Patient contact as part of postdischarge surveillance is usually through a questionnaire which is sent to patients through the post or completed during a phone call or in an outpatient setting.

However, there are some issues with usual care; traditional postdischarge SSI surveillance methods can be resource intensive, have been described as outdated and are not widely undertaken, while obtaining access to GPs can be difficult.^8 9^ These issues suggest a review of postdischarge wound follow-up is required; indeed, hospital staff involved in surveillance have called for modern digital approaches to surgical wound follow-up.^8^

Digital approaches to surgical wound follow-up have been partly facilitated by the COVID-19 pandemic which restricted patient travel and access to healthcare services.^10^ They are reported to show promise.^11^ Digital wound monitoring uses photos or online consultations to follow-up and assess patients’ wounds.^11^ However, patient engagement is essential for successful postdischarge surgical wound monitoring. A recent systematic review concluded engagement requires a greater understanding of the patients’ SSI experience.^12^

The aim of this study, therefore, is to explore patients’ and carers’ preferences for post-discharge surgical wound monitoring.

## Materials and methods

This is an explanatory mixed method design.^13^ A survey focused on issues relating surgical wound follow-up which were explored in more depth in a set of qualitative interviews. The survey was conducted between the 1 and 31 March 2022 with interviews undertaken until the 9 May 2022. Ethical permissions were approved by the East of Scotland Research Ethics Service (21/ES/0098).

### Patient and public involvement (PPI)

Three patient and public involvement (PPI) members, with lived experience, were recruited from existing local and national cardiothoracic PPI groups to join the research team. They were actively involved as co-applicants on the study: acquiring funding, commenting on the design, writing patient-facing material, agreeing the data themes and disseminating findings. One of the PPI members took on a PPI researcher role by undertaking data collection. Bespoke training in conducting interviews (tutorial and mock interview) was provided by one of the research team with qualitative research expertise.

### Recruitment and eligibility

Patients and carers were invited to participate in an online survey distributed via the Cardiothoracic Interdisciplinary Research Network and cardiac surgery PPI groups in London and Leicester, UK. London and Leicester were specifically included to maximise the opportunity to include participants from a diverse demographic population. Recipients were invited to share the survey link with other patients and carers. Inclusion criteria were adult patients having, or previously having had, any type of surgery or carers of adult patients having surgery. There was no restriction on the type of surgery as wound monitoring methods are generic across surgical specialisms in England. Exclusion criteria were people under 18 years old and people living outside England.

Participants completing the survey were invited to contact the research team if they wished to take part in a subsequent interview. Participants were eligible for inclusion in the subsequent interview if they had previously had any type of surgery, were over 18, able to give consent, spoke English or had someone who could translate and had access to Microsoft Teams. Having a previous wound infection or wound complication was not an inclusion criterion.

### Sample sizes

There were no restrictions on the survey sample size as we wished to gain an initial overview of surgical wound follow-up rather than achieve a statistically representative patient population sample. Interview sampling was pragmatic and dependent on survey respondents volunteering to participate. Data collection was guided by information power rather than reaching data saturation.^14^ Information power suggested a small sample, around six to 10 participants, and should generate sufficient data as the aim of study was narrow, the interview participants had specific experience, the quality of the interview data was high and there was a thematic approach to analysis.

### Survey

The survey was developed by the research team including the PPI members. It was distributed by the research team to 10 patients and carers for piloting, and subsequent amendments were made. The questions focused on participant demographics, SSIs and treatment, experiences of surgical wound follow-up and preferences for surgical wound follow-up. Most survey questions were closed with opportunities to provide additional free text where possible. The survey was hosted on JISC Online Surveys, and participation was voluntary and anonymous. Following piloting, additional questions were added regarding respondent demographics and pilot data were discarded.

### Interviews

The interview schedule was developed following analysis of the survey data and allowed for an in-depth exploration of issues identified through the survey (online supplemental material). Interviews were semistructured and focused on patients’ experiences of looking after their surgical wound at home and wound follow-up. Interviews were conducted online using Microsoft Teams either by an experienced researcher or by a trained peer (PPI) researcher, depending on availability. Following consent, the interviews which lasted around 1 hour were audio recorded with permission and independently professionally transcribed. The interview schedule was tested with two pilot interviews. Pilot interview data are included within the main interview data as no amendments were made to the schedule.

### Analysis

Quantitative survey data were analysed using simple descriptive statistics; qualitative data from the surveys and interview transcripts were analysed together using thematic analysis.^15^ Interview transcripts and qualitative survey data were read by a member of the research team experienced in qualitative research. The research team member then generated initial codes, driven by the data. Codes were reviewed and progressively refined into themes. A second researcher independently coded a sample of two transcripts to confirm the analysis reliability. We ensured rigour by checking the interpretation of data within the research team, feeding back findings to our PPI members (but not survey respondents) and through reflexivity.

## Findings

Seventy completed surveys were received. Seventy-four percent of survey respondents had undergone at least one surgical procedure, and although respondents were not statistically representative of all surgical patients, they broadly represent a wide range of ages, sex, ethnic groups and all regions of England. All six respondents who contacted the research team were interviewed. All six of the interviewees had previously had surgery with five developing an SSI. Demographics for the survey respondents and interview participants are shown in tables1  2 respectively.

**Table 1:** Survey participant demographics

Surveys	n=70
Age in years	
80–89	3 (4%)
70–79	10 (14%)
60–69	18 (26%)
50–59	16 (23%)
40–49	3 (4%)
30–39	15 (21%)
20–29	5 (7%)
Sex	
Male	31 (44%)
Female	39 (56%)
Ethnicity	
White British	41 (59%)
Asian/Asian British	26 (37%)
Black/African/Caribbean	3 (4%)
Location	
London	30 (43%)
Midlands	13 (19%)
North East and Yorkshire	8 (11%)
North West	6 (9%)
South West	6 (9%)
South East	5 (7%)
East of England	2 (3%)
Surgery	
Previously had surgery	52 (74%)
Cardiac surgery	15 (29%)
Non-cardiac surgery	50 (96%)
Not had surgery	18 (26%)

**Table 2:** Interview participant demographics

Interviews	6
Age	
80–89	2
70–79	1
60–69	3
Sex	
Male	5
Female	1
Ethnicity	
White British	5
Black/African/Caribbean	1

Data from the survey are presented within the themes generated from the qualitative interview data. The following themes were generated:

- The impact on patients of having an SSI.
- Patients’ preference for post-discharge surgical wound follow-up.
- Surgical wound monitoring using digital technology.
- Access to specialist support.
- Receiving information from the hospital about surgical wounds and wound care.
- Patients’ willingness to get involved in caring for their wounds.

### The impact on patients of having a surgical site infection (SSI)

Five of the six interviewees had an SSI with four being readmitted and having further surgery because of the infection. The interviewees described how developing a wound infection affected them physically, emotionally and financially, with readmissions and further surgery impacting their lives by taking months or years to heal and causing financial problems.

*I didn’t mix or socialise as much. I couldn’t go out because I was terrified of knocking it. It took months to heal*. (Participant 3)

*I had to go back for more surgeries to have the surgical site infection dealt with and that left me with an open wound which took around2yearsto heal. I went to the tissue viability clinic twice a week for2years. Here I am now about12yearson, and it’s not fully healed yet. I couldn’t work for2years…and I ended up going into debt and mortgage arrears. I have never recovered from that financially.* (Participant 4)

Being at home with a wound infection at home was ‘scary’, and interviewees felt ‘alone’ and ‘isolated’. They were worried or anxious and wanted reassurances about their wounds.

*What a scary thing it is to go home with a wound. Scary, and lonely. I could see it was starting to fester. I remember feeling really, really anxious about it. I felt lost because I didn’t really know where to go*. (Participant 3)

### Patients’ preference for postdischarge surgical wound follow-up

Of the 52 survey respondents who had experienced any surgery, 12 (23%) recalled the hospital following them up at home to ask about wound healing. Follow-up was either by telephone (50%), a home visit (25%), through an out-patient clinic (17%), by questionnaire in their discharge pack (17%) or by questionnaire through the post (8%).

When asked how likely they were to respond to requests for information about their wound by questionnaire around 1 month after surgery, survey respondents replied as follows (table 3). Most approaches were favourable, with emails and phone calls most likely to gain a response (96% and 94% respectively were definitely or probably willing to respond). While digital approaches were welcomed, the exception was digital approaches that involved having to download and instal an App. Only 37% of respondents would ‘definitely’ or ‘probably’ respond to an information request which involved downloading and installing an App, while a total of 50% said they ‘probably’ or ‘definitely’ would not.

**Table 3:** Survey respondents’ likely engagement with wound information requests*

Ways of completing questionnaire	Definitely	Probably	Don’t know	Probably not	Definitely not
In discharge pack	31%	33%	13%	19%	4%
In discharge pack with stamped addressed envelope	44%	37%	4%	11%	3%
With stamped addressed envelope received through the post	41%	34%	9%	13%	3%
Phone call at home	73%	21%	1%	0%	4%
Via email	57%	39%	3%	1%	0%
Via text message link	41%	39%	10%	9%	1%
Accessed on the internet via web browser	34%	41%	11%	10%	3%
Via downloaded and installed App	14%	23%	13%	34%	16%
Send photo securely via mobile phone	51%	31%	13%	1%	3%

* Not all percentage responses add up to 100% due to rounding.

The six interviewees received a variety of follow-up approaches after they were discharged from hospital. This included being instructed to contact the hospital or the GP if there was a problem, and, or invitations to postoperative clinic appointments.

*I went home and I was told, you know, if you’ve got any concerns, give us a ring and come back in2 weeks’time and we’ll have another look at it. So that was about the size of it*. (Participant 4)

Interviewees appreciated having a contact number if they had concerns.

*They have a clinical nurse specialist who I can get in touch with anytime. She might not be available. I might have to leave a message, but she’ll always get back. And I really like that.* (Participant 3)

However, despite being given contact phone numbers, two of the six interviewees were admitted to hospital as emergencies when their wounds opened up. These were distressing situations for the participants and their families.

*I was with my family on a weekend away and it was8 weeksafter the hysterectomy, so I was just coming out of recovery and this awful wound happened. I rang 999. And they were trying to assess the bleeding, you know,‘how many pads are you using?’. The paramedics said you need towels. We got into A&E, and I literally flooded the floor. I had to have a transfusion. It was a pretty dismal time*. (Participant 3)

When interviewees were asked what their preferred ‘no-expense-spared’ follow-up would be, a couple of participants said an initial home visit from a professional shortly after leaving hospital would be their preferred method if money were no object.

*A visit and then maybe a phone call. Few days later or a week later, just to check on how things are doing even.* (Participant 1)

### Surgical wound monitoring using digital technology

Issues surrounding remote digital technology were explored in the interviews as survey respondents viewed digital monitoring positively, and all six interviewees suggested this was their preferred approach to monitoring. The exception, however, were Apps that required to be downloaded and installed. Most interviewees embraced digital methods and spoke about how they liked video calls and sending the hospital pictures of their wounds.

*I think it’s brilliant (sending photographs). I’ve done that before*. (Participant 4)

*I’d prefer a video call because there are so many phone apps that you’ve got, and they take up a lot of memory.* (Participant 6)

*It was a novelty at the beginning, wasn’t it. But after COVID everybody’s got used to Zoom. I think most people my age will have used Zoom.* (Participant 3)

Indeed, healthcare professionals who did not take photos were perceived to be lagging behind.

*I said to the community nurse who came, why don’t you take a photograph of it and send it off to get it assessed. And she said we can’t do that because of data protection. I didn’t understand that. It seemed to me that the professionals hadn’t caught up with the value of being able to take a photograph.* (Participant 5)

A quick and professional response from the hospital to a digital submission was important.

*I just emailed it to her. She gave me an address that I could use. She picks it up, as I say, usually very quickly, which I think is a very important thing as well. I wouldn’t want to send [my photo] into a black hole and nobody looks at it. I would expect to hear back within24hours*. (Participant 1)

A key reason for the preference for digital monitoring was the impact on resources. Travelling to hospitals for clinic appointments or to the GP was expensive, inconvenient and perceived as wasting time for both patients and staff.

*We don’t need to waste time travelling to visit the hospital. If you go into hospital now you can easily lose half a day just sitting waiting.* (Participant 2)

*Not only does [sending photos] save me a lot of time‘cos I don’t have to go to the surgery or the hospital, but it saves the medical team a lot of time as well.* (Participant 4)

*[Showing somebody a photograph] is much easier than having to go back to the hospital to visit a clinic, to show them your wound. You know the transplant unit is60–70miles away, the hip was done 50 miles away, so yes, it’s better than driving all the way up there.* (Participant 1)

Most of the interviewees, both male and female, appeared to have a laid-back approach to image security. They were either not concerned about security or there was a perception that National Health Service (NHS) sites were secure, and they trusted NHS staff with their images.

*It was an NHS address, so I presume it’s fairly secure. I don’t really bother about [security].* (Participant 1)

*[Security] didn’t occur to me. Perhaps it should because I was stripped to the waist and my face was on it. I should have excluded my face.* (Participant 5, male)

*I didn’t have too many concerns about [security]. I have to trust the medical profession*. (Participant 4)

Most of the interviewees, who were in their sixties to eighties, thought some people might struggle with technology, especially older people, and would need alternative methods of communication.

*People of my age group seem to be reasonably comfortable using it, but then there’s one or two that won’t.* (Participant 1)

*That’s the age group that we have to be most concerned about. I would say probably 70 upwards, 75 upwards.* (Participant 1)

*If you say you don’t use the internet, then someone could just phone you.* (Participant 2)

When asked how frequently they would like to share images of their wounds with the hospital, interviewees felt this could be daily initially, to provide reassurance, or that the frequency should be dictated by the clinician.

*Well, I think I’d probably go on your advice. If you want me to send you a photograph every couple of days, I will. It’s really about what you feel you need from me.* (Participant 1)

*I would say perhaps for the first four or5 days, do it daily and then, wean it off because it’s the early days that are the worst*. (Participant 3)

When engaging in wound care follow-up, most of the interviewees discussed how their partners or other close family members assisted them in taking photos of their wound.

*I tried to take the picture by reversing the camera. I managed to take pictures of everything but the wound. So, I asked my wife to do it.* (Participant 2)

### Access to specialist support

Support was not always available outside office hours and two interviewees who needed help with their wounds over the weekend had to visit an emergency department. One interviewee discussed how reduced services and expertise at the weekend in the emergency department and also on the wards meant there were no skilled staff available.

*We ended up going to [the Emergency Department]‘cos it was a Saturday. And there was nobody in [the Emergency Department] that knew anything about vacuum dressings at all. And when they tried phoning the ward to get somebody down to deal with replacing the vacuum dressing, there was nobody on duty that could deal with it*. (Participant 4)

Accessing healthcare support was compounded by difficulties accessing GPs.

*I might have tried to get in to see the GP, but it wasn’t easy to do that.* (Participant 5)

*I can walk to my GP, it’s that close, but getting an appointment is a whole different ball game.* (Participant 1)

Interestingly, some interviewees were reluctant to contact the hospital or their GP when they were concerned about their wound. Several spoke about not wanting to ‘bother people’ and ‘taking up [staff] time’. On occasion, this meant interviewees delayed seeking advice or treatment, possibly allowing their wounds to worsen, even if they had been instructed to do this.

*They asked me to get in touch if I am worried, but I will leave it a few days to see if it gets any worse. They’re really busy. I don’t want to bother them. On the ward I saw them running up and down, they were rushed off their feet*. (Participant 2)

One interviewee who was seen by a practice nurse suggested there was an element of not wanting to ‘step on toes’ between acute and primary care.

*My practice nurse is really good with wounds. I was hoping that she would be able to look after the wound but she didn’t want to touch it because I was connected to the hospital.* (Participant 3)

### Receiving information from the hospital about surgical wounds and wound care

Most survey respondents (92%) said they would like to receive information about caring for their wound and the signs and symptoms of infection at the point of discharge from hospital. Respondents were asked to select their top three preferred options, from a suggested list, for receiving such information. Responses were by hospital leaflet (73%), followed by discussion with a hospital nurse (69%), photo of their wound sent to the hospital (50%), discussion with the surgeon (39%) and in a video about wound monitoring (25%). The least popular options were a UK Health Security Agency leaflet (11%), poster displayed in hospital (4%), podcast about wound healing (6%) and Facebook page (4%). Interviewees said there was a lot of information being given in the hospital which some found reassuring, but others found overwhelming. Having information in a written format was helpful as this could be revisited if needed.

*When you go in you get bombarded with a tremendous amount of information which to be honest is not helpful.* (Participant 2)

*I got a leaflet. But the fact that I didn’t really have to use it means that I didn’t really retain it. It was probably reassuring at the time.* (Participant 1)

If they needed to talk with a healthcare professional about their wound, the interviewees felt that a nurse would be more knowledgeable about wound healing or approachable than a surgeon.

*I think the nurse can give you a much clearer, better understanding of how the wound is doing*. (Participant 1)

Interviewees were used to obtaining information online and thought a website would be a good forum for information about wound infections, with a trusted site being helpful.

*I did some searching on the internet before I went in. You have trusted sites where you can get information. So [professionals] can give some information preoperatively*. (Participant 2)

Key information wanted by interviewees was about normal wound healing and practical advice on how to care for their wound.

*It’s a bit of a mystery really what [the wound] should look like.* (Participant 3)

*A video [about normal wound healing] would be useful.* (Participant 2)

*And then you know what to do about it, washing it down with the saline solution,for example, being more careful about personal hygiene in that area, etc.* (Participant 4)

Some interviewees said they knew their own bodies and could sense when something was not right.

*You get to know your own body, especially when different things start to go wrong.* (Participant 6)

I *was worried about it. I knew it wasn’t quite right.* (Participant 3)

In these situations, when interviewees suspected something was wrong with their wound, they wanted to have images on a website of normal wound healing to help reassure them.

*People can look at [photos on a website] and say oh I’m normal and then move on.* (Participant 4).

### Patients’ willingness to get involved in caring for their wounds

As well as demonstrating their engagement with wound monitoring by taking photographs of their wounds, the interviewees also displayed a willingness to become involved in managing and treating their wounds.

*[Digital photos] would be good to teachpatients. You could just change a dressing at home yourself and send in a picture of it.* (Participant 2)

*When I got home it opened slightly, but I got in touch with the nurse and she said to strap it with those steri-strips you get. She said to put five or six across the wound, so I did, and that worked perfectly well.* (Participant 1)

*They had managed to train my other half to do the dressings. The deal was that I could go home and she would change dressings daily, and then twice a week I would come into the tissue viability clinic.* (Participant 4)

## Discussion and conclusion

This study explores patients’ and carers’ experiences of, and their preferences for, postdischarge surgical wound monitoring.

Wound healing was problematic for patients, with 10% of the survey respondents who had surgery including four of the six interviewees being re-admitted to hospital because of their wound infections. Although patients can contact their hospital or GP if they have a concern, some participants in the study were reluctant to ‘bother’ hospital staff which delayed them seeking advice. While there was no reported reluctance to contact GPs, participants, along with many other people in the UK, reported experiencing difficulties in obtaining GP appointments.^16^ Interviewees echoed the experiences of patients in other studies when they spoke about feeling isolated and there being a lack of support when they were discharged home with a surgical wound.^17^ They also spoke about considerable financial loses and wound treatments that lasted for years. Readmissions, difficulties in accessing healthcare, plus the experiences described by some of the interviewees suggest there is room for improvement within post-discharge surgical wound monitoring. A new approach to wound monitoring that is proactive and ongoing should reassure patients and be able to identify wound problems early so treatment can start before problems deteriorate.

Most participants supported digital approaches for communicating with healthcare staff about their wounds. Digital approaches to wound monitoring are becoming more widely used following the COVID pandemic.^10^ Studies show, or suggest, digital surgical wound monitoring reduces readmissions, reduces GP and emergency department visits, is accurate, has high patient compliance, high patient satisfaction and can reduce carbon emissions.^1118^,^21^ Survey respondents’ top preferences for follow-up at 30 days after surgery were via telephone, email and sending photos, while interview participants said sending photos of their wounds and video calls were their preferred methods. Although wound monitoring platforms that required software to be downloaded and installed were the least favoured survey option. Participants in this study said quick responses to their wound concerns, providing reassurance and having easy access to health services with a point of contact were important to them. These attributes are all provided within digital monitoring.^18^ Interviewees felt websites showing images of normal wound healing would help provide further reassurance.

Although most interviewees were in their 60s, 70s or 80s and liked digital monitoring, they thought that increasing age could be a barrier to participating in digital monitoring. Though this might be overcome by an ‘assisted’ approach to wound monitoring where patients who are unable to use digital technology are assisted by a relative or carer who is able.^18^ Indeed, the interviewees described the involvement of their own partners in their wound care through attending meetings, taking photographs and changing dressings. While a small number of recent studies have identified patients’ ‘positive anticipation’ to get involved in wound monitoring,^22 23^ a theme emerged during the interviews where interviewees discussed their willingness to take on wound care responsibilities. Willingness to become more involved in caring for and treating their wounds is a new finding that has the potential to reduce NHS clinician workload and improve clinical outcomes ultimately reducing NHS (and patient) costs. While patients show interest in self-wound care this requires further exploration of its feasibility and safety.

PPI in this study was beneficial, especially in undertaking interviews where the PPI researcher engaged easily with interviewees and opened up new avenues of inquiry. The number of survey respondents was not sufficient to provide generalisable data; however, this was not the aim of the survey. The aim was to identify issues for further investigation. A limitation of using a survey was that respondents were limited by the options presented. Although only a comparatively small number of participants volunteered to be interviewed, the interview data was substantial and rich in explanatory power, and it identified valid issues relating to postdischarge surgical wound monitoring. Therefore, the interview data is the main focus of this paper.

In summary, this study finds that better surgical wound monitoring after hospital discharge is needed. Digital approaches to surgical wound monitoring are preferred by patients. More work exploring the wide scale implementation of digital surgical wound monitoring, including the cost and resource implications, and creating trusted patient information websites would be beneficial. Both survey respondents and interviewees displayed a willingness to participate in wound monitoring, and interviewees demonstrated an existing involvement in wound care management. Future investigations could explore how best to involve patients further in surgical wound care monitoring and management.

## supplementary material

10.1136/bmjopen-2024-087320 online supplemental file 1

## Data Availability

Data are available upon reasonable request.
