# Supplementary material for: Exploratory study of patients’ and carers’ preferences for postdischarge surgical wound monitoring using survey and interviews
Source: BMJ Open. 2025 Jan 25;15(1):e087320. doi: 10.1136/bmjopen-2024-087320 (PMC11784326; doi:10.1136/bmjopen-2024-087320)
Supplement: online supplemental file 1 [file bmjopen-15-1-s001.docx]

**Participant Interview Guide**

Topics for discussion

- Details of previous surgery
- Wound complications arising from previous surgery
- Knowledge of wound infections prior to surgery
- Experience of wound care follow-up
- Preference for wound care follow up
- Information provided by the hospital at discharge on caring for wounds
- Preference for wound information
