# Supplementary material for: Exploratory study of patients’ and carers’ preferences for postdischarge surgical wound monitoring using survey and interviews
Source: BMJ Open. 2025 Jan 25;15(1):e087320. doi: 10.1136/bmjopen-2024-087320 (PMC11784326; doi:10.1136/bmjopen-2024-087320)
Supplement: Supplementary file 2 [file bmjopen-2024-087320.reviewer_comments.pdf]

PEER REVIEW HISTORY

BMJ Open publishes all reviews undertaken for accepted manuscripts. Reviewers are asked to complete a checklist review form and are provided with free text boxes to elaborate on their assessment. These free text comments are reproduced below.

ARTICLE DETAILS

Title (Provisional)

An exploratory study of patients' and carers' preferences for post-discharge surgical wound monitoring using survey and interviews

Authors

Tanner, Judith; Brierley Jones, Lyn; Westwood, Nigel; Rochon, Melissa; Wloch, Catherine; Rogers, Luke J; Vaja, Ricky; Dearling, Jeremy; Wilson, Keith; Harrington, Pauline; Brown, Colin S; Murphy, Gavin

---

VERSION 1 - REVIEW

**Reviewer**	**1**
**Name**	**Gledhill, Kate**
**Affiliation**	**Monash University, Faculty of Medicine, Nursing and Health Sciences**
**Date**	**18-Jun-2024**
**COI**	**Nil competing interests**

Thank you for the opportunity to review this manuscript. Addressing patient and carer preferences in healthcare is essential to developing models of care that are more effective, so I commend you on addressing this. However, there are some concerns that need addressing prior to publication.

Introduction:

This introduction outlines and defines SSI well and gives an overview of the current model of care however more detail would help to identify the gap in the literature. Is there any evidence of issues in the current process? Greater depth of detail would make the gap in literature clearer to the reader. Currently, there is not a strong link to justify the aims.

Material and methods:

It is to be commended that you acknowledge the use of patient researchers however more detail is needed as to their involvement. What was the level of involvement (in particular in relation to design, was this participatory or consultive as an example) It is mentioned in data

collection that trained peer researchers, but more detail is needed on the training, how and who were they trained by?

Results:

The demographic details are well presented and clear, I commend you on the breadth of participants. More clarity is needed however in the presentation of the themes. There are relationships and interactions that are not currently well explored and would add depth and clarity to your results. One example would be that there is an interaction between the impact of SSI and experience if SSI, I wonder if presenting these in reverse order would show the interaction between them. Some of the results presented in patient experience seems to fit better with service issues ie inadequate staffing on weekends and access to GP's. I wonder if there is a bigger theme here and these are the subthemes that fit under this. Some of the data under patient experience seems to link to the use of digital technology. There also appears to be a link between information delivery and the willingness to get involved. An exploration of these links and further refinement of themes would strengthen these results

Discussion and conclusion:

Greater analysis is needed to place the results of this study within the current literature. More detail is needed regarding the types and locations of existing literature. For example does any of the literature used in the digital technology discuss the accuracy or facilitators and barriers to the use of photographs?

Overall, I commend you for addressing issues that may reduce hospital readmissions, this is an essential focus in the current healthcare climate.

**Reviewer**	**2**
**Name**	**Tobiano, Georgia**
**Affiliation**	**Griffith University, CRE Wiser Wound Care**
**Date**	**28-Jun-2024**
**COI**	**Nil**

Thank you for the opportunity to review this well-written paper on an important topic: preferences for post-discharge surgical wound monitoring. Addressing the following feedback could enhance the manuscript:

Larger feedback:

For the following sentence: “Data collection was guided by information power (all the questions were answered) rather than reaching data saturation [13].” How was information power determined to be sufficient/what techniques used to ensure information power? Usually, convenience sampling requires larger samples for qualitative methods.

An explanatory mixed method design was used. The methods lacks details about how qual and quant data were integrated both at the data collection steps, and then during data analysis. It makes it tricky to review the results without these details, as it is not clear how the results were integrated and why they have been presented as they have.

For the survey more details required– how developed? Valid/reliable? Types of questions? Response options?

Minor feedback:

The introduction has a couple of short paragraphs/lone sentences – which interrupts the flow for the reader. For example “”Patient engagement is essential for successful post-discharge surgical wound monitoring, but, as a recent systematic review concluded, this requires a greater understanding of the patients’ SSI experience [11].”” Is a lone sentence – can this be incorporated into your argument in other paragraphs?

“Patient researchers were involved in the study design, acquiring funding, data collection, analysis and dissemination of findings.” More details about these patient researchers would strengthen the study (i.e. number of researchers, how recruited, type of lived experiences). Consider adding the GRIPP-2 checklist to guide additional details to provide for the reader.

Inclusion criteria not explicit, only exclusion criteria explicit for the reader in the following, please revise:

“Patients and carers were invited to participate in an online survey distributed via the Cardiothoracic Interdisciplinary Research Network (CIRN) and cardiac surgery Patient and Public Involvement (PPI) groups in London and Leicester, UK. London and Leicester were specifically included to maximise the opportunity to include participants from a diverse demographic population. Recipients were invited to share the survey link with other patients and carers. While the initial focus was cardiac surgery, adult patients having any type of surgery and carers were eligible to complete the survey as wound monitoring methods are generic across surgical specialisms in England. Exclusion criteria were people under 18 years old and people living outside England. “

In the design, inductive approach is highlighted. But it looks like inductive approach was only used for data analysis? Consider removing from overall study design.

Thematic analysis used, please provide more details about the steps in this process.

VERSION 1 - AUTHOR RESPONSE

Responses to reviewers’ comments

**Comment**	**Response**
**Reviewer 1**	
This introduction outlines and defines SSI well and gives an overview of the current	This is a really helpful comment, thanks. We have added a new paragraph

model of care however more detail would help to identify the gap in the literature. Is there any evidence of issues in the current process? Greater depth of detail would make the gap in literature clearer to the reader. Currently, there is not a strong link to justify the aims.	identifying some of the issues with current usual care which make the argument that we need to consider other (digital) approaches to post-discharge wound follow -up
Material and methods:<br>It is to be commended that you acknowledge the use of patient researchers however more detail is needed as to their involvement. What was the level of involvement (in particular in relation to design, was this participatory or consultive as an example) It is mentioned in data collection that trained peer researchers, but more detail is needed on the training, how and who were they trained by?	Thank you. We have added some more detail to this section.
Results:<br>The demographic details are well presented and clear, I commend you on the breadth of participants. More clarity is needed however in the presentation of the themes. There are relationships and interactions that are not currently well explored and would add depth and clarity to your results. One example would be that there is an interaction between the impact of SSI and experience if SSI, I wonder if presenting these in reverse order would show the interaction between them. Some of the results presented in patient experience seems to fit better with service issues ie inadequate staffing on weekends and access to GP's. I wonder if there is a bigger theme here and these are the subthemes that fit under this. Some of the data under patient experience seems to link to the use of digital technology. There also appears to be a link between information delivery and the willingness to get involved. An exploration of these links and further refinement of themes would	You suggested we consider swapping the order of the Impact SSI and the Experience of wound follow-up. I tried this, but the flow of the overall sequence of the themes did not work well. So, I have kept Impact of SSI as the first theme. I have removed some data from Patients preferred method of follow up and inserted it into the Experience of wound follow up theme and renamed the theme Preference for wound follow-up. As suggested, I have moved the digital monitoring related material out of Patients preferred method of follow up and merged it into the Digital theme. As suggested, I have created a new theme – Access to specialist support. I looked at linking Information delivery and Willingness to get involved, but not see a link between them.

strengthen these results	
Discussion and conclusion:<br>Greater analysis is needed to place the results of this study within the current literature. More detail is needed regarding the types and locations of existing literature. For example does any of the literature used in the digital technology discuss the accuracy or facilitators and barriers to the use of photographs?	I have listed the benefits of digital wound monitoring. I have not discussed all the issues surrounding digital monitoring as this is not the main focus of this paper.
**Reviewer 2**	
For the following sentence: "Data collection was guided by information power (all the questions were answered) rather than reaching data saturation [13]." How was information power determined to be sufficient/what techniques used to ensure information power? Usually, convenience sampling requires larger samples for qualitative methods.	I have added more information to the methods sample size section explaining information power for the interview participants.
An explanatory mixed method design was used. The methods lacks details about how qual and quant data were integrated both at the data collection steps, and then during data analysis. It makes it tricky to review the results without these details, as it is not clear how the results were integrated and why they have been presented as they have.<br>For the survey more details required– how developed? Valid/reliable? Types of questions? Response options?	Thank you, hopefully this is now clearer.<br>I have added information to the Interview section describing how the interview schedule was developed from the survey data.<br>I have added information to the Analysis section to make it clear that quants data from the survey was analysed with simple descriptive stats, while quals data from the survey and interviews were analysed together using thematic analysis.<br>I have added more detail to the Survey section.
The introduction has a couple of short paragraphs/lone sentences – which interrupts the flow for the reader. For example ""Patient engagement is essential for successful post-discharge surgical wound monitoring, but, as a recent systematic review concluded, this requires a greater understanding of the patients' SSI experience [11]."" Is a lone sentence – can this be incorporated into your argument in other paragraphs?	I have amended this sentence slightly and incorporated it within a paragraph.

"Patient researchers were involved in the study design, acquiring funding, data collection, analysis and dissemination of findings." More details about these patient researchers would strengthen the study (i.e. number of researchers, how recruited, type of lived experiences). Consider adding the GRIPP-2 checklist to guide additional details to provide for the reader.	I have added some more information about the PPIs. I have not provided detail about their personal lived experiences as their names are listed as co-authors and they are therefore identifiable.<br><br>The paper includes information on most of the GRIPP 2 short form topics. It does not include any reflections or critical perspectives as our PPI co-applicants, including the PPI researcher, are writing a paper about their experiences of taking part in the study which includes this information and we wish to avoid duplication.
Inclusion criteria not explicit, only exclusion criteria explicit for the reader in the following, please revise:<br>"Patients and carers were invited to participate in an online survey distributed via the Cardiothoracic Interdisciplinary Research Network (CIRN) and cardiac surgery Patient and Public Involvement (PPI) groups in London and Leicester, UK. London and Leicester were specifically included to maximise the opportunity to include participants from a diverse demographic population. Recipients were invited to share the survey link with other patients and carers. While the initial focus was cardiac surgery, adult patients having any type of surgery and carers were eligible to complete the survey as wound monitoring methods are generic across surgical specialisms in England. Exclusion criteria were people under 18 years old and people living outside England. "	I have rewritten this to make the inclusion criteria clearer.
In the design, inductive approach is highlighted. But it looks like inductive approach was only used for data analysis? Consider removing from overall study design.	I have removed any references to an inductive approach
Thematic analysis used, please provide more details about the steps in this process.	I have added more information to the Analysis section.

VERSION 2 - REVIEW

Reviewer 1

Name Gledhill, Kate

Affiliation Monash University, Faculty of Medicine, Nursing and Health Sciences

Date 20-Dec-2024

COI

Thank you for so thoroughly addressing the revisions suggested. This is a much improved paper and I am satisfied that the main concerns were addressed.

I have a few last recommendations.

In the introduction, paragraph 3 sentence 1 is unclear, consider editing for clarity.

Material and methods section paragraph 2 you have sufficiently outlined the PPI involvement, however one further clarification you could provide would be to clarify what commenting on analysis means.

You may benefit from including a brief summary of what amendments were required following the pilot testing of the survey, were these amendments minor for clarity or were there bigger concepts that were added/ removed etc?

In analysis section, was there any member checking completed or just with the PPI members?

The first quote under access to services theme, you have changed A&E to emergency department in the first sentence but not the second, consider changing this for clarity.

Overall though all initial recommendations were addressed.

Thank you for your attention to detail

VERSION 2 - AUTHOR RESPONSE

Responses to reviewers' comments

Resubmission 2

Comment	Response
**Reviewer 1**	
In the introduction, paragraph 3 sentence 1 is unclear, consider editing for clarity.	I have revised this sentence to make it clearer.

Material and methods section paragraph 2 you have sufficiently outlined the PPI involvement, however one further clarification you could provide would be to clarify what commenting on analysis means.	I have replaced 'commenting on analysis' to 'agreeing the data themes'.
You may benefit from including a brief summary of what amendments were required following the pilot testing of the survey, were these amendments minor for clarity or were there bigger concepts that were added/ removed etc?	I have updated the sentence to say that 'following piloting, additional questions were added regarding respondent demographics and pilot data were discarded'.
In analysis section, was there any member checking completed or just with the PPI members?	I have amended the sentence to read 'We ensured rigour by checking the interpretation of data within the research team, feeding back findings to our PPI members (but not survey respondents) and through reflexivity.
The first quote under access to services theme, you have changed A&E to emergency department in the first sentence but not the second, consider changing this for clarity.	Both now read 'Emergency Department'
**Reviewer 2 – no additional comments**	
